# Neurological Emergencies in Incarcerated Patients: Clinical Characteristics, Severity, and Outcomes in an Emergency Department with an Embedded Neuro-Emergency Expert Model

**DOI:** 10.3390/brainsci15101069

**Published:** 2025-09-30

**Authors:** Byung Joon Choi, Jin Hyouk Kim, Won Soek Yang, Young Sun Park, Sang Ook Ha

**Affiliations:** 1 Department of Emergency Medicine, Hallym University College of Medicine, Anyang 14068, Republic of Korea; dltackw@naver.com (B.J.C.); wsyang@hallym.or.kr (W.S.Y.); ndyspark@hallym.or.kr (Y.S.P.); 2 Department of Neurology, Hallym University College of Medicine, Anyang 14068, Republic of Korea; neubrain@hallym.or.kr

**Keywords:** incarceration, emergency department, neurological emergencies, functional neurological disorder, metabolic encephalopathy, stroke

## Abstract

**Background**: Incarcerated patients with neurological complaints present substantial diagnostic and care-delivery challenges in emergency departments (EDs). We delineate the clinical spectrum, severity, and outcomes among incarcerated patients managed in an ED with an embedded neuro-emergency expert model. **Methods**: A retrospective observational study of adult ED visits for neurological symptoms was conducted from September 2018 to June 2025 at a government-designated regional emergency center serving multiple correctional facilities. Incarceration was confirmed in the electronic medical record. Extracted variables included demographics, chief complaint, comorbidities, triage and acuity scale, Glasgow Coma Scale (GCS), neuroimaging, ED diagnoses, and outcomes (hospital admission, ICU care, ED/in-hospital mortality). **Results**: Sixty-five patients were included (median age 57.0 years [IQR 47.0–64.5]; 95% male). Chief complaints were altered mental status (36.9%), hemiparesis (21.5%), and seizures (13.8%). On arrival, 40.0% had GCS ≤ 12, including 23.1% with severe impairment (GCS 3–8). Non-contrast head CT was obtained in 95.4% and diffusion-weighted MRI in 38.5%. Frequent diagnoses were psychiatric/functional neurological disorder (16.9%), metabolic encephalopathy (15.4%), and acute ischemic stroke (12.3%). Serious conditions (stroke, hypoxic brain injury, central nervous system infection, status epilepticus, and neuroleptic malignant syndrome) were diagnosed in 41.5%. Hospital admission occurred in 63.1% (ICU care in 47.7%); in-hospital mortality was 10.8%. **Conclusions**: ED visits by incarcerated individuals with neurological complaints were often linked to serious diagnoses, ICU use, and mortality, challenging assumptions of exaggeration. Over two in five had stroke, hypoxic brain injury, central nervous system infection, or status epilepticus. The findings support rapid, systematic, bias-aware evaluation with early neurological involvement, clear imaging triggers, safety protocol, and expedited transfers from correctional facilities.

## 1. Introduction

Neurological symptoms are common reasons for emergency department (ED) visits, ranging from mild dizziness and headaches to life-threatening conditions such as stroke, status epilepticus, and meningoencephalitis [1]. Rapid and accurate assessment is essential yet challenging, even for experienced clinicians. In incarcerated patients, these challenges are amplified by unique health issues, limited access to care, and potential provider bias [2]. Globally, incarcerated populations bear a disproportionate burden of infectious diseases, chronic conditions, and mental illness compared to the general population [3,4]. In South Korea, a nationwide study found a markedly higher prevalence of major diseases among inmates; for example, diabetes mellitus was approximately six times more common and depression about forty-seven times more common than in the general population [5]. These health disparities often lead to complex clinical presentations when acute illness occurs.

ED management of incarcerated patients is further complicated by structural, social, and psychological factors. Security procedures can delay timely care, and provider bias may negatively influence diagnostic and treatment decisions when symptoms are considered malingered or drug-seeking [2]. Neurological complaints in incarcerated patients are often stereotyped as secondary gain behaviors; medically unexplained or non-organic presentations, including functional neurological disorder (FND), are therefore frequently encountered in correctional settings—a pattern also noted in the ED literature [6]. Such bias risks delaying the diagnosis of a true neurological emergency.

Correctional facilities have high burdens of mental disorders (e.g., depression) and physical illnesses (e.g., infectious diseases); epilepsy is also prevalent [4]. In addition, the congregate nature of prisons is associated with a markedly elevated tuberculosis burden [7]. In Korea, men constitute about 92% of inmates, and the incarcerated population is aging; older inmates often have multiple comorbidities that exceed on-site correctional medical capacity, prompting more hospital transfers [8,9]. Despite these trends, studies on ED care and inpatient management of incarcerated patients with neurological symptoms are lacking.

To the best of our knowledge, this is the first report in South Korea to specifically identify neurological emergencies within the incarcerated population, addressing a clear gap created by limited prior data on ED treatment for this group. Leveraging our institution’s Neuro-Emergency Medicine Program—an ED-embedded neuro-emergency expert (NEE) model (NEM) that provides immediate specialist input [10]—we analyzed the clinical characteristics, diagnostic pathways, and early management of incarcerated patients presenting with neurological symptoms. In alignment with the conclusions of our study, the authors sought to interpret these issues and their outcomes to clarify the clinical and systems-level significance of timely, bias-aware, specialist-led evaluation and to inform pragmatic improvements in ED care for incarcerated patients.

## 2. Materials and Methods

### 2.1. Identifying Participants and Cases

Adult patients (18 years and over) who visited the ED with neurological symptoms between September 2018 and June 2025 were screened. Electronic medical records (EMR) were searched for the keywords “prison” or “incarceration”, and documents such as guard escort notes and other security-related records were reviewed to confirm incarceration status. Repeat visits for the same neurological event were excluded.

### 2.2. Role of the Neuro-Emergency Expert and ED Workflow

**Figure 1:**
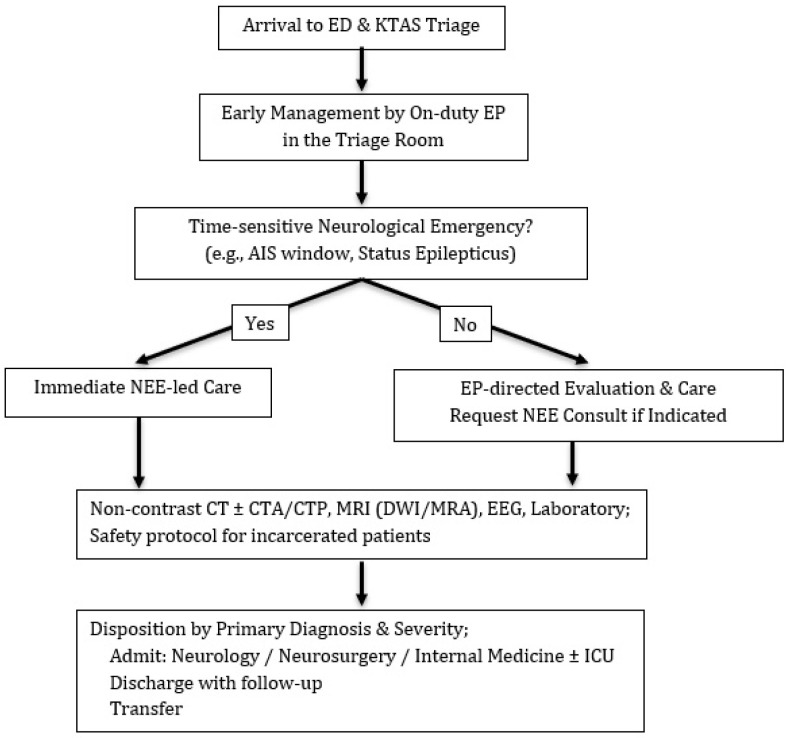
Standard neuro-ED workflow.

Our emergency department operates a dedicated NEM project. The neuro-emergency expert is a board-certified neurologist who obtained certification 22 years ago and has served as an attending in the emergency department for more than a decade. The expert supervises weekday care from 08:00 to 18:00. Upon arrival, patients with neurological complaints are triaged with the Korean Triage and Acuity Scale (KTAS) [11]. An on-duty emergency physician (EP) initiates early management in the triage room. The NEM expert’s primary responsibilities include direct care of patients presenting with typical neurological conditions in the ED and consultation for patients primarily admitted under other services (e.g., internal medicine). Patients who arrive within time-sensitive treatment windows, such as acute ischemic stroke or status epilepticus, receive immediate NEE-led management. Patients with non-time-sensitive neurological symptoms are initially managed by EPs, with NEM consultation requested when clinically indicated. Depending on the principal diagnosis, patients are admitted to Neurology, Neurosurgery, or Internal Medicine (ICU vs. ward) or are discharged/transferred as appropriate (Figure 1).

### 2.3. Data Collection and Variables

Variables extracted from the EMR using a standardized case report form included age, sex, and comorbidities (hypertension, diabetes mellitus, dyslipidemia, prior ischemic or hemorrhagic stroke, coronary artery disease, atrial fibrillation, chronic kidney disease, cirrhosis, malignancy, and seizure). Data were also collected on major neurological complaints, Glasgow Coma Scale (GCS) score on arrival, Korean triage and acuity scale (KTAS) level, neuroimaging studies, and outcomes (hospital admission, ICU admission, length of stay, and death). Chief complaints were categorized based on the documented findings of the triage nurse or emergency physician. “Altered mental status” was broadly defined to include confusion, decreased responsiveness, drowsy, stupor, and coma. Based on the emergency physician’s assessment, the neuro-emergency expert finalized the ED diagnosis and grouped it into categories: Serious diagnoses included stroke, hypoxic brain injury, central nervous system infection, status epilepticus, and neuroleptic malignant syndrome. If multiple conditions were present, the primary diagnosis was taken as the main cause of the presentation (e.g., uremic encephalopathy due to renal failure was classified as metabolic encephalopathy).

### 2.4. Ethical Considerations

The study was conducted in accordance with the Declaration of Helsinki and approved by the Institutional Review Board of Sacred Heart Hospital of Hallym University (IRB No.: 2025-08-016)5. Given the retrospective design, the use of anonymized data, and the low risk to participants, the institutional review board waived the need for informed consent.

### 2.5. Statistical Analysis

Categorical variables are expressed as number (%) and continuous variables as medians with interquartile range (IQR). Comparative statistical tests were not performed due to the small sample size and the descriptive nature of the study. Statistical analysis was performed using IBM SPSS Statistics version 26.

## 3. Results

### 3.1. Baseline Characteristics

A total of 65 incarcerated patients with neurologic complaints were included (Table 1). The median age was 57.0 years (IQR 47.0–64.5). Individuals in their 40s and 50s accounted for more than 60% of the cohort; none were over 80 years old, and about 17% were under 40 years. Most patients were male (*n* = 62, 95%). Hypertension was the most common comorbidity (*n* = 24, 36.9%), followed by diabetes mellitus (*n* = 23, 35.4%) and dyslipidemia (*n* = 11, 17.2%). Chronic kidney disease was present in 10 patients (15.4%), including 4 on dialysis. Malignancy (9.2%), liver cirrhosis (4.6%), coronary artery disease (4.6%), and atrial fibrillation (4.6%) were also observed. A history of seizure was recorded in nine patients (13.8%). Prior ischemic and hemorrhagic stroke histories were documented in eight (12.3%) and six (9.2%) patients, respectively. Psychiatric diagnoses were not routinely documented in the ED records due to limitations, but some patients had known histories (e.g., schizophrenia spectrum disorder: four cases; panic disorder: one; bipolar disorder: one). 

### 3.2. Neurological Severity, Imaging, and Chief Complaints

As shown in Table 2, the median GCS score on arrival was 15. However, among the 65 patients, 26 (40.0%) had moderate-to-severe impairment on ED arrival (GCS ≤ 12), including 15 (23.1%) with severe scores (GCS 3–8) and 11 (16.9%) with moderate scores (GCS 9–12); the remaining 39 (60.0%) had mild scores (GCS 13–15). KTAS triage levels were as follows: stage 1 (21.2%, *n* = 14), stage 2 (37.9%, *n* = 25), and stage 3 (40.0%, *n* = 26). Most patients received rapid diagnostic evaluation. A non-contrast brain CT was performed immediately in 62 of 65 patients (95.4%), revealing acute lesions in approximately one-third of these cases. Additional imaging included CT angiography (CTA) in four patients (6.2%), with perfusion CT in one case and multiphase CTA in two cases. Diffusion-weighted MRI was performed in 25 patients (38.5%) and combined DWI and MR angiography in 6 patients (9.2%). Electroencephalogram (EEG) was performed in seven patients (10.8%).

**Figure 2:**
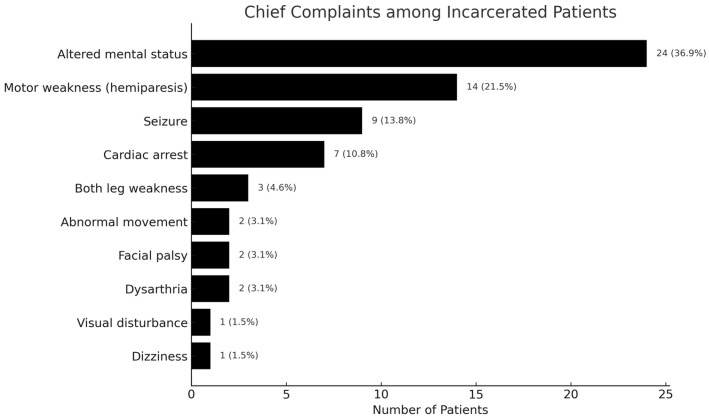
Chief complaints of incarcerated patients presenting to the ED.

As shown in Figure 2, altered mental status was the most frequent chief complaint (24 patients, 36.9%), followed by hemiparesis (14 patients, 21.5%) and seizures (9 patients, 13.8%). Seven patients (10.8%) were found unresponsive in the correctional facility and arrived in cardiac arrest after receiving pre-hospital CPR; of these, three were self-hanging attempts. Other complaints included both leg weakness (three patients, 4.6%), abnormal movements (two, 3.1%), facial palsy (two, 3.1%), dysarthria (two, 3.1%), visual disturbance (one, 1.5%), and dizziness (one, 1.5%).

### 3.3. Emergency Department Diagnosis

**Figure 3:**
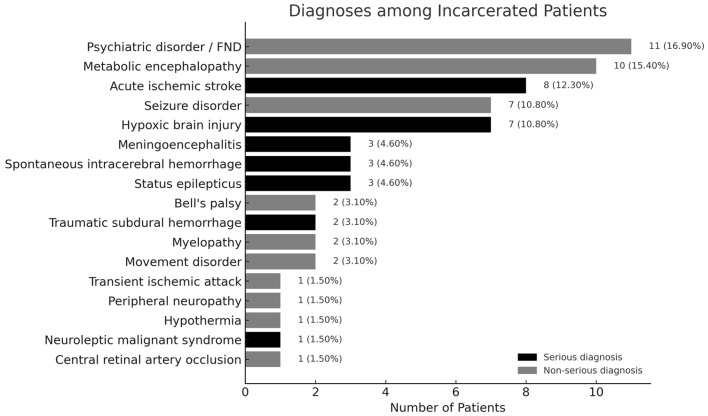
ED diagnoses among incarcerated patients with neurological complaints.

As shown in Figure 3, the most frequent single ED diagnosis was psychiatric disorders/FND (11 patients, 16.9%), followed by metabolic encephalopathy (10, 15.4%) and acute ischemic stroke (8, 12.3%). Serious diagnoses (black bars in the figure) were made in 27 patients (41.5%) and included acute ischemic stroke; hypoxic brain injury (7, 10.8%); central nervous system infection (3, 4.6%); spontaneous intracerebral hemorrhage (3, 4.6%); status epilepticus (3, 4.6%); traumatic subdural hemorrhage (2, 3.1%); and neuroleptic malignant syndrome (1, 1.5%). Non-serious diagnoses (gray bars; total 38 patients, 58.5%) included psychiatric disorders/FND, metabolic encephalopathy, seizure, myelopathy (2, 3.1%); movement disorders (2, 3.1%); Bell’s palsy (2, 3.1%); and single cases (1.5% each) of transient ischemic attack, peripheral neuropathy, hypothermia, and central retinal artery occlusion.

### 3.4. Clinical Outcomes

Emergency room management and disposition: Endotracheal intubation was performed in 10 patients (15.4%). After ED management, 41 patients (63.1%) were admitted: 17 (26.2%) to neurology, 17 (26.2%) to internal medicine, and 7 (10.8%) to neurosurgery. One patient with hypoxic brain injury (1.5%) died in the ED, twenty-one (32.3%) were discharged home from the ED, and two (3.1%) were transferred to another hospital.

In-hospital course: Among admitted patients, 31 (47.7%) required ICU care (75.6% of those hospitalized). In-hospital mortality occurred in seven patients (10.8%). The median hospital length of stay was 8.0 days (IQR 4.0–16.0) and median ICU length of stay was 4.0 days (IQR 3.0–12.3) (Table 3).

## 4. Discussion

In this single-center cohort of 65 incarcerated patients presenting with neurologic complaints, we observed a predominantly middle-aged male population with substantial vascular or metabolic comorbidity, arriving with high acuity (40% GCS ≤ 12; 59.1% KTAS 1–2). Altered mental status, hemiparesis, and seizures were the leading presentations, and an immediate CT-first strategy (95.4%) yielded acute lesions in approximately one-third, with MRI/vascular imaging used selectively. Diagnoses spanned both serious organic disease (41.5%; e.g., acute ischemic stroke, hypoxic brain injury, ICH, CNS infection, status epilepticus) and non-serious/functional or psychiatric etiologies, underscoring the need for rapid discrimination pathways. Resource utilization and risk were high (intubation 15.4%, admission 63.1% with ICU use in nearly half; in-hospital mortality 10.8%).

We identified several notable patterns in this vulnerable population with implications for emergency care and public health. First, incarcerated patients typically present with more severe neurological symptoms than the general ED population. This likely reflects a selection bias in transfers from correctional facilities to hospitals and delayed help-seeking under carceral conditions. In our cohort, about 40% had at least moderate impairment of consciousness. Compared with our prior report in the general ED population, the symptom distributions underscore this acuity gap. In the general ED, the most common chief complaints were dizziness (31.8%), hemiparesis (24.2%), altered consciousness (15.8%), and seizures (8.7%) [10]. By contrast, in the present cohort, altered mental status predominated (36.9%), followed by hemiparesis (21.5%) and seizures (13.8%); dizziness was rare (1.5%). Minor or nonspecific complaints are seldom reported from correctional facilities and are often managed on-site; hospital transfer tends to occur only after clear deterioration. Early reporting of neurological symptoms by incarcerated individuals appears to be uncommon, possibly due to concerns about being perceived as malingering or skepticism regarding the likelihood of appropriate care. Accordingly, emergency physicians should treat neurological presentations in incarcerated individuals as high-risk, with systematic and expedited evaluations.

Second, the diagnostic spectrum in the incarcerated population differed clearly from that of the general ED population. The overall rate of serious diagnoses was higher in the present cohort (41.5%) than in the general ED cohort (31.7%) [10]. Diagnoses reflecting systemic illness and infection were comparatively more frequent (metabolic encephalopathy 15.4% vs. 6.9% and CNS infection 4.6% vs. 1.0%). Seizure-related diagnoses were also increased, with seizures (10.8% vs. 7.4%) and status epilepticus (4.6% vs. 1.4%). Beyond this higher diagnostic yield in the ED, management gaps specific to prison settings have been highlighted: in a UK prison cohort, Thomasson et al. reported limited access to specialist epilepsy services (only 38.2% had a specialist review within the prior 12 months), and most incarcerated individuals with epilepsy (76.4%) did not recall receiving guidance about occupational duties or cell arrangements appropriate to their diagnosis [12]. By contrast, peripheral vertigo, a typical low-acuity presentation, accounted for only 1.5% in the present cohort versus 24.1% in the general ED; AIS was lower (12.3% vs. 19.8%), and cerebral hemorrhage was similar (7.7% vs. 7.1%). Meanwhile, psychiatric disorders/FND represented at 16.9% in the present cohort, markedly higher than the 0.4% observed in the general ED. These differences may reflect the high psychosocial stress inherent to correctional environments or possibly that some inmates report symptoms as a means to temporarily leave the facility. Nevertheless, clinicians should not prematurely dismiss incarcerated patients’ neurological complaints as malingering. In this study, all FND diagnoses were confirmed only after comprehensive testing had excluded organic causes, underscoring the need for a balanced, systematic diagnostic approach that remains alert to bias.

Third, metabolic encephalopathies were common. This finding reflects deficiencies in chronic disease management within correctional facilities. Examples in our study include uremic encephalopathy in patients with chronic kidney disease, toxic encephalopathy from drug overdose, encephalopathy resulting from delayed infection treatment, and hypoglycemia. These emergencies appear largely preventable with timely and appropriate management. Limitations in chronic disease care in correctional settings have been consistently reported [3,4,5,13]. Implementing routine chronic disease management and formal correctional–hospital care pathways may directly reduce neurologic emergencies. Telemedicine is a promising strategy to expand specialist access and facilitate early intervention in resource-limited environments [14].

Fourth, the stroke rate was relatively low in this cohort. Contributing factors may include the relatively young average age of inmates, under-recognition of mild stroke symptoms in the facility, and delays in transfer. Incarcerated patients diagnosed with stroke received care comparable to the general population, in accordance with international guidelines; specifically, intravenous thrombolysis (*n* = 1) and endovascular thrombectomy (*n* = 1) were administered. Given evidence that implicit bias can affect clinical outcomes, regular bias recognition training is recommended [15]. Future efforts should include stroke awareness training for correctional staff and standardized rapid transfer protocols.

Finally, outcomes in incarcerated patients depend heavily on the timeliness and appropriateness of treatment. In the general ED cohort, 55% were admitted, 41.8% were discharged to outpatient follow-up, and in-hospital mortality was 0.3% [10]. By contrast, in the present cohort, 63.1% required admission, nearly half required ICU care (47.7%), and in-hospital mortality reached 10.8%, underscoring greater acuity and complexity. With a dedicated neuro-emergency expert embedded in our ED, our model delivers timely diagnosis and management across the spectrum of neurological emergencies, including time-critical cases. Hospitals without such systems may face a greater risk of delay. All emergency department staff should recognize the specific vulnerabilities of incarcerated patients and manage them as a high-risk population, with streamlined activation pathways, bias-aware triage, and early specialist involvement.

From a public health perspective, health issues in incarcerated individuals extend beyond prison walls. Most incarcerated people eventually return to their communities, and gaps in correctional medicine can have community-wide impacts. Infectious diseases, untreated mental illness, uncontrolled epilepsy, and other chronic conditions can pose lasting risks after release. Therefore, improving correctional health care is not only a humanitarian obligation but also a key preventive strategy for community health and safety [16,17].

## 5. Limitations

This was a retrospective, single-center study with a small sample restricted to inmates from local correctional facilities who presented to one regional emergency center; therefore, generalizability is limited. The observational, nonexperimental design precludes causal inference, and interventional/functional testing lay outside the scope and logistics of the embedded NEM project. Although we benchmarked our data against published general ED neurology cohorts, the absence of a contemporaneous, matched non-incarcerated control group—and potential differences in case mix and practice patterns across studies—limits cross-cohort comparability. In addition, our embedded NEM model likely improved recognition and timeliness of care; therefore, comparisons with centers lacking on-site neuro-emergency expertise may not be directly comparable, potentially inflating diagnostic yield and affecting outcomes. The carceral context may also have introduced selection and information biases, and the small sample size reduces precision and constrained subgroup analyses. Future larger, multi-center studies with standardized data collection are needed to better characterize neurological emergencies in incarcerated patients and to develop evidence-based guidelines that integrate correctional care, emergency medicine, and neuro-emergency expertise.

## 6. Conclusions

In this study, ED visits by incarcerated individuals with neurological complaints were frequently associated with serious diagnosis, ICU care, and mortality, countering the bias that their symptoms are exaggerated or non-organic. Although psychiatric/FND cases were present, more than two in five patients had serious conditions such as stroke, hypoxic brain injury, central nervous system infection, or status epilepticus. Accordingly, a rapid, systematic, and bias-free evaluation is essential. In the ED, care should include early neurological involvement, clear imaging triggers, safety protocols for managing incarcerated patients, and expedited transfer pathways coordinated with correctional facilities. In addition, strengthening chronic disease management within correctional settings can reduce preventable emergencies, and incarcerated patients should receive acute neurological care that is as equitable and rigorous as that provided to non-incarcerated patients.

## Figures and Tables

**Table 1:** Baseline characteristics of incarcerated patients with neurological emergencies.

Variable	Enrolled Patients (N = 65)
Demographics	
Age (years)	57.0 (47.0–64.5)
18 ≤ age < 40	11 (16.9%)
40 ≤ age < 60	24 (36.9%)
60 ≤ age < 80	30 (46.2%)
Sex	
Female	3 (4.6%)
Male	62 (95.4%)
Major Comorbidities	
Hypertension	24 (36.9%)
Diabetes mellitus	23 (35.9%)
Dyslipidemia	11 (17.2%)
Chronic kidney disease	10 (15.4%)
Seizure disorder	9 (13.8%)
Ischemic stroke	8 (12.3%)
Hemorrhagic stroke	6 (9.2%)
Malignancy	6 (9.2%)
Coronary artery disease	3 (4.6%)
Atrial fibrillation	3 (4.6%)
Liver cirrhosis	3 (4.6%)

Data are presented as number (percentage) or median (25–75th percentile).

**Table 2:** Clinical severity and diagnostic evaluation in the ED.

Variable	Enrolled Patients (N = 65)
Clinical severity	
Glasgow Coma Scale on ED arrival	
Severe (GCS 3–8)	15 (23.1%)
Moderate (GCS 9–12)	11 (16.9%)
Mild (GCS 13–15)	39 (60.0%)
KTAS level	
1	14 (21.2%)
2	25 (37.9%)
3	26 (40.0%)
Diagnostic modality	
Computed tomography	
Brain non-contrast CT	62 (95.4%)
Brain CT with CTA	4 (6.2%)
Brain CT with perfusion CT	1 (1.5%)
Brain CT with multiphase CTA	2 (3.1%)
Magnetic resonance imaging	
Brain DWI only	25 (38.5%)
Brain MRA + DWI	6 (9.2%)
Electroencephalogram	7 (10.8%)

Data are presented as number (percentage). CT, computed tomography; CTA, computed tomography angiography; DWI, diffusion-weighted imaging; ED, emergency department; GCS, Glasgow Coma Scale; KTAS, Korean Triage and Acuity Scale; MRA, magnetic resonance angiography.

**Table 3:** ED disposition and in-hospital outcomes for incarcerated patients.

Variable	Enrolled Patients (N = 65)
Endotracheal intubation	10 (15.4%)
Emergency department disposition	
Admission to hospital	41 (63.1%)
Admission to Neurology ward	17 (26.2%)
Admission to Internal Medicine	17 (26.2%)
Admission to Neurosurgery ward	7 (10.8%)
Discharged from emergency department	21 (32.3%)
Transfer to another hospital	2 (3.1%)
Mortality in emergency department	1 (1.5%)
In-hospital admission	
Admission to intensive care unit	31 (47.7%)
Length of stay in hospital, days	8.0 (4.0–16.0)
Length of stay in intensive care unit, days	4.0 (3.0–12.3)
Mortality in hospital admission	7 (10.8%)

Data are presented as number (percentage) or median (25–75th percentile).

## Data Availability

No new data were created or analyzed in this study. Data sharing is not applicable to this article.
